# Implications of Argentine Tango for Health Promotion, Physical Well-Being as Well as Emotional, Personal and Social Life on a Group of Women Who Dance

**DOI:** 10.3390/ijerph18115894

**Published:** 2021-05-31

**Authors:** Joanna Witkoś, Magdalena Hartman-Petrycka

**Affiliations:** 1 Faculty of Medicine and Health Science, Andrzej Frycz Modrzewski Krakow University, 30-705 Kraków, Poland; 2 Department of Basic Biomedical Science, Faculty of Pharmaceutical Sciences in Sosnowiec, Medical University of Silesia, 41-200 Sosnowiec, Poland; mhartman@sum.edu.pl

**Keywords:** Argentine tango, dance, women’s health, physical well-being, social life, menstrual cycle disorders

## Abstract

Background: The aim of the research was to determine the effect that dance has on the promotion of health, physical well-being, as well as the emotional, personal and social life of women who dance. In addition, the impact of the physical activity of long, often all-night dancing events on women’s health was investigated. This included possible disturbances in their monthly cycle and circadian rhythm, taking into account symptoms of biological rhythm disturbances. Methods: The study involved 214 women: tango group: 109, sedentary group: 105. The Mann–Whitney U and chi^2^ tests were used to compare the groups, as well as multiple ordinal regression to analyse individual predictors of missed menstrual periods. Results: The tango vs. sedentary groups did not differ in the duration of menstrual bleeding, the degree of pain during menstruation, the regularity of menstruation, the number of regular monthly cycles per year, and amenorrhea. Intermenstrual spotting was more common in dancers (tango 12.8% vs. sedentary 4.8%; *p* = 0.038). The frequency of missed periods was not increased by any of the assessed aspects. In 59.6% of female dancers, milongas caused disturbances in circadian rhythms, including extreme fatigue and drowsiness (36.7%), 66.0% of the dancers mentioned only positive aspects of Argentine Tango’s impact on their personal life. Conclusions: tango plays a positive and multifaceted role in the lives of dancers and fulfils the need for social contact. The physical effort put into this form of physical activity does not significantly affect the menstrual cycle, and thus the reproductive functions, and can be recommended as an attractive and safe form of physical recreation for women.

## 1. Introduction

Dance and music occupy an important place in the history and social structure of every world culture. The universality of dance lies in the fact that there is no culture or society in the world that does not practice it in some form. It is a fascinating subject to study, but all too often little attention is paid to it and it rarely appears in scientific literature. Dance is defined as a way of expressing human emotions through a specific artistic movement [1,2,3].

In recent years, art, music and dance have become important topics when considering the influence of artistic expression on the promotion of health in both sick and healthy people. Recognition of the benefits of using artistic resources, such as dance therapy, for the well-being and health of people, is definitely growing [4,5,6]. Dance, in particular, has been suggested as an excellent example of an activity that combines physical exercise with emotional involvement. It is mainly due to these features that research into the possible applications of dance in health care was developed. It has long been known that physical activity has a positive effect, not only on physical, but also, on mental health. In dance, social interactions, pleasure, relaxation and artistic expression are combined with an attention to physical fitness. According to the World Health Organization (WHO), physical activity is one of the cornerstones of a healthy lifestyle, especially in an ageing population. People of all ages and cultures around the world seem to enjoy movement and dancing in response to music, especially rhythmic stimulation [7]. In addition to the above, dance is also perceived as a way of spending free time, of familiarising oneself with the culture of another country and last, but not least, of making new social contacts. Dancing skills used to be an important element of a good upbringing as well as social good manners. Nowadays, these skills can elevate social standing and reflect certain socially welcomed skills that symbolise the achievement of a higher status of personal development [8].

Dance is the ‘art of conversation’ between partners, a nonverbal reading of the partner’s intentions and then compliance with these intentions. A particular case of such a dialogue and nonverbal dance communication is the Argentine tango (AT), danced in pairs with unwritten but binding rules of etiquette and good manners. AT is a complex phenomenon which includes music, dance, partner relationships and lifestyle. AT has not undergone standardisation, which, in practice, means that it does not have one universal model, and, this, in turn, contributes to its attractiveness. The dancers bring their own style and ‘decorations’ to the tango. Although these are based on learned techniques, they are presented according to the dancer’s own personality, which greatly increases the enthusiasm and unpredictability of the dance experience. AT, as an important element of Latin American culture, has come a long way. From the niche dance of the African slaves, through the dance culture of Latin America, it entered the UNESCO list of intangible cultural heritage in 2009 [1,2,3].

Nowadays, the tango has become more iconic than ever before. There is an increased interest in this dance, fully supported in Argentina and Uruguay as a symbol of their heritage. Practiced by hundreds of thousands of people around the world, it captivates an international audience as it spreads across national borders, helping to establish new relationships between different nations in a time of accelerating globalisation [9,10,11,12,13,14].

Physical activity is a key predictor of human health and a manifestation of a lifestyle associated with a specific system of values, not only practising sports in the narrowest sense the word. Available research results indicate a strong correlation between undertaking physical exercise, especially aerobic training, and better mental functioning, both cognitive and emotional. Systematic physical exercise promotes increased blood flow through the brain, which in turn provides nutrients and oxygen. It also regulates the stimulation of the sympathetic nervous system and the reactivity of the hypothalamic-pituitary-adrenal axis, promotes neurogenesis and ensures the correct level of neurotransmitters in the brain, including serotonin, dopamine, endorphins and norepinephrine [5]. In addition, dance activity improves cognitive abilities such as space-time orientation and synchronised movement with a partner. Dancers want to do more than just learn the steps, they want to do them gracefully and stay in synchronisation and harmony as a couple [6,7]. It has been shown that people who do aerobic training have a greater volume of both grey and white matter in the prefrontal and temporal cortex than those who are sedentary. This may be due to increased oxygenation in the prefrontal cortex, which is observed in exercising people after just a few days of exercise [7].

Dancing, particularly, compared to other forms of physical recreation, forces dancers to correct their body posture as they perform to make their profile more attractive, enforces better eye-hand co-ordination and smooth movements. Physical activity contributes to an improvement in the parameters of the cardiovascular system and a reduction in the resting and exercise blood pressure and leads to an increase in physical capacity and an improvement in exercise tolerance. In addition, it has a positive effect on changes in the lipid profile, improves glucose tolerance, and prevents the development of osteoporosis [15]. Movement improves general well-being and helps to reduce depression thanks to endorphins released into the body during physical activity [16].

It is known that increased physical activity can lead to female athlete triad, described in scientific literature [17,18]. As the relationship between the availability of energy, i.e., the often unconscious maladjustment of the body’s energy needs to the level of energy expenditure, and physical activity. This may even include the occurrence among physically active women, especially professional sportswomen, of eating disorders (anorexia, bulimia) and the disappearance of the menstrual cycle and osteoporosis [17,18]. It is well known that achieving a good result in any competition requiring physical effort is associated not only with many hours of daily training, but, also, with optimal body weight for that sport discipline. In the case of sports such as: artistic gymnastics, swimming, figure skating and also typical artistic activities such as ballet or dance, consistently maintaining a slim figure is a necessity. That is why many women who train desire or need to reduce body weight, in order to improve the effectiveness of their exercises, the physical efficiency of their body and the aesthetics of the movements performed during, for example, dancing.

AT dancing is a multidimensional experience affecting many aspects of the personal and social life as well as the physical and mental health of the dancer. According to McKinley [19] AT dancing influences six main areas important for a better quality of life: physical exercise, social satisfaction, spirituality and focus, emotional health, learning, and thus enhancing knowledge. In addition, it has been proven that these areas improve and develop in both healthy and disabled people [6]. Emotional reactions, invoked by music and strengthened by dance, influence specific brain activity connected with auditory-motor integration, which additionally points to the positive influence of dance on the human body [19]. 

The aim of this study was to learn about the impact of dance, namely AT, on health promotion, physical well-being, as well as on the emotional, personal and social life of dancing women. In addition, the impact of the physical activity of long, often all-night dancing events on women’s health was investigated. This included possible disturbances in the monthly cycle and the circadian rhythm, taking into account symptoms of biological rhythm disturbances.

## 2. Materials and Methods

### 2.1. Participants in the Study

The study involved 109 women aged 22 to 51 years (mean ± standard deviation: 37.3 ± 6.6 years) who attended Argentine tango classes and events and 105 women from a control group (sedentary group) aged 23 to 54 years (36.0 ± 7.1 years), who declared that they did not regularly participate in sport. The full profile of the women is presented in Table 1. The control group consisted mainly of students from one of the universities in Kraków and women who were either their family and/or friends.

There were significant differences in body weight, interpreted as body mass index (BMI) value, between the group of tango-dancing women and the physically inactive women from the sedentary group. The average median BMI in the dancing women was 20.8, while in the physically inactive women it was 24.0 which is close to the border for the overweight range (Table 1).

The study excluded women who were not experiencing a menstrual cycle due to pregnancy, menopause, or another factor not directly related to exercise, e.g., hysterectomy. In the control group, women who declared regularly practising sports for more than one hour per week were also excluded. An incorrectly completed questionnaire also resulted in exclusion from the research. Participation in the research was voluntary and anonymous and, in accordance with the Declaration of Helsinki, the participants were informed of the purpose of the research and the option to refuse to answer the questionnaire. The research protocol was reviewed and approved by the Bioethical Committee of the Andrzej Frycz Modrzewski Krakow University (Permission number KBKA/93/O/2020).

### 2.2. Survey

The author’s own survey was used for the research. The survey was conducted on-line, through Internet portals intended for AT dancers. The survey was created using Google Forms. For the control group, the questionnaire was distributed using the network of contacts of the university students. The questionnaire included questions about the number of hours a woman spent dancing tango, its impact on her monthly cycle and the circadian rhythm. Additionally, questions were asked about the motivation to undertake this type of physical activity and the impact it had on the personal lives of the dancers. The survey used in the research is included as Appendix A.

### 2.3. Statistical Analysis

Statistical analysis was performed using the SPSS 21 software program (Version 27.0, IBM Corp., Armonk, NY, USA). Three types of analyses were performed. The first one presents the descriptive results of all data in total. In the second, the groups of women dancing AT and the control group were compared. The third examined the correlations and predictors of menstrual disorders.

Means, medians, standard deviations as well as minima and maxima were used to describe the numerical data (continuous) and for dichotomous and qualitative data-numbers and percentages. The comparison of both groups for numerical variables was carried out using the Mann–Whitney U test, and for dichotomous and qualitative ones: the chi-square test. Correlations were calculated using Spearman’s rho correlation coefficients. Multiple ordinal PLUM (Polytomous Logit Universal Model) regression analyses were used to analyse the predictors of menstrual disorders and predictors of disturbances in circadian rhythm after milongas. Results of *p* < 0.05 were considered statistically significant.

## 3. Results

### 3.1. Involvement in Dance

The commitment to dancing of the AT dancers surveyed was as follows: on average, they had been dancing tango for seven years, six hours a week and, by the end of the research, they had taken part in twelve marathons or festivals, with seven of them involving many hours of travelling to other cities or even countries. Additionally, on average, they participated in eight workshops lasting four hours (Table 2). 72 dancers, i.e., 66.1% of the group answered the question “Have you taken breaks from the intensity of tango dancing?” affirmatively.

### 3.2. Characteristics of the Menstrual Cycle

The analysis showed that there were no significant differences in the duration of menstrual bleeding and the degree of pain on these days in the dancers compared to the control group. Additionally, no differences were observed in the general regularity of monthly cycles and the number of regular cycles occurring in one calendar year. There were also no differences in the exercise-induced loss of monthly cycles and the frequency of using hormonal contraception (Table 3 and Table 4).

The only significant difference in the course of monthly cycles in tango-dancing women compared to physically inactive women was the higher incidence of intermenstrual spotting (tango 12.8% vs. sedentary 4.8%) (Table 4).

In order to analyse the predictors of the disappearance of the menstrual cycle for the answers to the question ‘Have you ever had any disturbances in the menstrual cycle, if so, for how long?’, the following numeric codes were assigned: never: 0; less than 3 months: 1; between 3 and 6 months: 2; over 6 months: 3. Firstly, the correlations between the disturbances in the menstrual cycle and all the predictors, as well as between the predictors themselves, were calculated. Very few associations were found between the predictors and disturbances in the menstrual cycle. The only significant correlation was with BMI: the higher the BMI, the longer the disappearance of the menstrual cycle (R = 0.14, *p* < 0.05) (Appendix B). Multiple ordinal PLUM regression with logit binding function was performed. Three of the independent variables: the number of years dancing, the number of marathons and festivals, and the number of workshops were quite highly correlated with each other. Since high correlations between predictors are unfavourable for multiple regression analysis, only one of these three variables, namely the number of years of AT dancing, was used. The assumption of equality of the regression coefficients for each predictor in each cumulative category of the dependent variable (each independent variable has an identical effect at each cumulative split of the ordinal dependent variable), which is important for ordinal regression, was satisfied: chi^2^(14) = 17.15; *p* = 0.249. The general goodness-of-fit test yielded a statistically insignificant result chi^2^(7) = 3.73; *p* = 0.811. This means that all predictors combined did not affect the dependent variable. Nagelkere’s pseudo-R-square value was very small (0.04). None of the independent variables studied, namely BMI, number of years of tango dancing, number of hours of tango per week, disturbances in circadian rhythm after milongas, trips to a milonga away from the place of residence and taking breaks from the intensity of tango, turned out to be a statistically significant predictor of disturbances in the monthly cycle (Appendix C). 

### 3.3. Influence of Dance on the Dancers’ Circadian Rhythm 

Participation in milongas in 59.6% of dancers contributed to the disturbance of the circadian rhythm, mainly: extreme fatigue and drowsiness (36.7%), nonrestorative sleep (29.4%), inability to focus (20.2%) and difficulties in falling asleep and staying asleep (16.5%) (Table 5). 

Multinomial logistic regression for the dependent variable occurrence/nonoccurrence of circadian rhythm disturbances after a milonga and independent variables: age, BMI, as well as the number of years of tango dancing, number of hours of tango per week, trips to a milonga away from the place of residence and taking breaks from the intensity of tango have not shown significant relationship either, at R-square Nagelkerke: 0.08 (Appendix D).

### 3.4. Influence of Tango on Personal Life

Answers to the open-ended question ‘Please briefly describe if and how tango dancing has influenced your personal life’ were as follows: 66.0% of the dancers mentioned only positive aspects of AT’s impact on their personal life, 9.2% indicated both positive and negative changes, 6.4% emphasised only a negative influence, and 18.3% did not respond to this question. The percentage of people whose answers were classified into a specific category of life changes is presented in Table 6.

## 4. Discussion

Hundreds of thousands of people in most regions of the world now dance AT and the number of people choosing this type of physical activity to meet their various personal needs is steadily increasing. Olsewski [18] claims that, the popularity of tango around the world is largely due to its authenticity. Participation in many hours of milongas, festivals or marathons, often taking place in different countries of the world, has become an intercultural experience combined with the possibility of ‘touching’ a foreign culture, something which is unknown, exotic and exciting. For many AT amateurs, dance is a way of spending their free time, a milonga is a place to make new friends and a distraction from everyday life and real problems. Tango connects people who share the same passion and interest in the aesthetics and technique of this dance, representing a kind of subculture [8,17].

The appeal of this physical recreation, which is AT dancing, makes the women in our dancing study group practice many hours over many years. They participated in milongas, marathons and festivals which often involved long journeys to another city or country. In addition, they took part in workshops lasting up to four hours. Considering, therefore, the physical energy needed in the dance and the time that the women spend on dancing AT, travelling, festivals, and marathons, it makes sense to investigate whether they expose themselves to unfavourable health problems which occur in women who undertake too much physical activity.

### 4.1. Influence of Dance on the Dancers’ Menstrual Cycle

Our research showed that despite the considerable effort that dancing women put into AT dancing, including participation in long-lasting milongas and marathons, often involving long journeys, this did not disturb their monthly cycle. The analysis showed that the dancers, compared to the control group, did not differ significantly in the duration of menstrual bleeding, the degree of pain experienced during these days, the regularity of the monthly cycles, or the occurrence of secondary amenorrhoea. It is worth emphasising that in the studied group, a detailed analysis of factors directly related to dance, that could affect the menstrual cycle, did not reveal any key factor which determined the occurrence of disorders, and this may have been expected due to the high physical effort exerted in training. The only significant difference in the course of monthly cycles in AT-dancing women compared to physically inactive women was the increased incidence of spotting between menstruation (tango 12.8% vs. sedentary 4.7%). There are many reasons for the occurrence of intermenstrual spotting, but the most frequently indicated is incorrect secretion of steroid hormones by the ovaries. Scientific literature [19,20] shows that both functional hypothalamic amenorrhea and intermenstrual spotting can develop as a result of intense physical activity or severe stress. Perhaps this spotting among the studied dancers is the first symptom which could indicate the possibility of future disorders of the menstrual cycle caused by AT dancing. However, female athlete triad [21] is not relevant in this case, because secondary amenorrhoea did not take place.

### 4.2. Influence of Dance on the Dancers’ Circadian Rhythm

In our research, some women stated that AT dancing significantly disturbed their circadian rhythm. Many scientific publications [22,23,24] describe the influence of external factors on the disturbance of the natural biological rhythm, which controls and regulates most of the processes taking place in the body. Milongas and tango marathons always take place during the evening and at night and this certainly leads to physiological disturbances in the dancers’ hours of sleep. Sleep is an essential element of the circadian cycle as well as of the process of regeneration and reconstruction of the body. A lack of sleep contributes to the occurrence of a number of symptoms that indicate a bodily malfunction. The negative health effects of sleep deprivation include tiredness, confusion, headaches and an inability to concentrate. It seems that disturbances in the circadian rhythm are quite strongly connected with AT dancing, and nocturnal gatherings are not conducive to the proper regeneration of the body.

This is confirmed by the results of our own research, as 59.6% of the dancers declared that milongas caused disturbances, mainly extreme fatigue and drowsiness (36.7%), nonrestorative sleep (29.4%), inability to focus (20.2%) and difficulties in falling asleep and staying asleep (16.5%) in their circadian rhythm. However, it should be noted that in the general survey question, in which the dancers were free to comment on the impact of AT on their personal lives, only 10.1% answered that they slept less and had disturbed circadian rhythms. Undoubtedly, the aspect of AT related to circadian rhythm disturbances is not important enough for dancers to give up this dancing and its characteristic nightlife. It is worth emphasising that the disturbances in the circadian rhythm did not translate into the disappearance of the monthly cycle, which is a very important observation.

### 4.3. Influence of Dance on the Dancers’ BMI

Dance is a form of physical recreation which, as our own research has shown, can be part of a lifestyle and affect the current and future psychophysical health of dancers. It is also the basic determinant of physical fitness and therefore people should embrace it regardless of age and gender. An active way of spending free time plays an important role in preventing overweight and obesity, as was indicated by the BMI of the dancers in our own research. The average BMI of the dancing women was 20.8, while for the physically inactive women it was 24.0, which is approaching the overweight range limit of 25.0. The BMI of the dancers is healthy and may indicate the positive influence of dance on their body. It also improves the general psychophysical condition of the human body, facilitates recovery from the negative emotional states and mental tensions of everyday living. Correct body weight is an important factor in the prevention of many lifestyle diseases, such as diabetes, atherosclerosis, heart attacks and cancer [25,26,27].

In the case of AT dancing, there are no typical features of training goals, such as, reducing the level of adipose tissue in the body, because certain criteria, such as measuring the heart rate would have to be met in order to determine the heart rate necessary for effective fat burning. Despite this, it was, however, shown that the BMI of the dancing women was mostly normal. This, in turn, means it can be assumed, with a high degree of probability, that, when dancing women have maintained the same BMI over the years, other physiological parameters of the body have improved or will improve, thus affecting the maintenance of good health. Nowadays, overweight and obesity have taken the form of an epidemic and are among the common factors influencing the development of cardiovascular and metabolic diseases, so, dancing seems to be an excellent means of preventing these problems. Health benefits, better care of health and body as well as greater energy were observed by 23.9% of people who responded.

It cannot be excluded that the better BMI values obtained in the author’s own research may also result from the fact that part of the women in a specific city, will not be interested in attending tango activities at all, for instance. It means that only slim women with a naturally lower BMI will come to the milongas, for example. It should also be borne in mind that, in modern culture which promotes a slim figure, obese women may feel unattractive; therefore, those women’s participation in milongas will be limited. Obese women may also have health problems and limited physical fitness and, as a result, they may avoid attractions involving exercise. Obviously, the above does not rule out the supposition of a beneficial effect of tango on the body composition; it can be observed that the minimum BMI value in dancers is higher than in the control group, which may, however, be caused by the development of muscle mass promoted by exercise.

### 4.4. Influence of Dance on the Dancers’ Personal/Social Life

Dance is a form of social activity, rooted in human culture, that combines two expressions of human existence: physicality (the body and the ability to communicate through the body) and the psychological aspect (mind). Dance combines what is individual, intimate, bodily, empathetic and emotional with that which is social.

Physical activity, including dance, is an important activity that should be fostered in the community, because it allows an individual to identify themselves with a group with similar interests and goals and reduces social isolation, especially of lonely people. This leads to the achievement of social satisfaction, as our own research has shown. For many women (21.1%) an improvement in their social life was an important aspect of the changes associated with AT dancing.

The tango dancing community creates a harmonious and pleasant environment, offering numerous beneficial effects combining physical activity with art. Additionally, dance stimulates the ability to learn a new activity and contributes to a feeling of ‘connection and understanding’ of something exotic and so far unknown [28,29]. The dancers feel that they belong to a group of people with additional, attractive skills, which significantly distinguishes them from the rest of society, thus giving them a sense of distinction. Everyone involved in AT dancing, generally has their own clearly defined personal goal for doing so. It may be the desire to meet new friends and create new social interactions, or to learn something exotic. Dancers often find a passion in dance that improves their well-being, thus allowing them, during dance events, to ‘break away’, from their often depressing reality.

AT dancing is a social activity in which embodied knowledge, intimacy, empathy, a relationship between the sexes and the individual’s self-realisation all occur, expressed against a background of group dynamics. Our research has shown the multifaceted positive impact of this physical recreation, dancing, on the daily life of the respondents.

Considering AT as a ‘dance of the senses’ in which the grip between partners restricts the possibility of individual movement makes for a study of social interactions. The partners submit to and accept the agreement, which is subject to a very strict system of rules. This dance, therefore, has a very personal character and is of huge significance not only for the dancers who perform it, but also for the entire dancing community [30]. The dancing couple must sustain the harmony in a very unpredictable and undetermined choreography of their own kind of ‘dialogical embodiment’, which is one of the main tenets of AT. The unpredictability of events in the dance may have a real impact on the perception of reality in the everyday life of the dancers [30].

An important feature that distinguishes AT from other ballroom dances is that its steps are not difficult, it is actually a ‘walk with a partner’ [31,32,33]. Therefore, this dance is easy to assimilate, learn and dance compared to many other complicated ballroom dances. This encourages and attracts people of all ages, socioeconomic status and levels of fitness. So, dancing the tango is a pleasant and attractive social activity that can significantly improve quality of life and, additionally, it is accessible to everybody who knows and understands the steps, rules and etiquette of the dance.

### 4.5. Influence of Dance on the Dancers’ Travelling

Milongas are dance events which take place during the evening/night where the AT is danced. Participation in such events requires some prior preparation from the dancers, knowledge of the steps and the applicable conventions, as well as appropriate attire, including dance shoes. Despite the fact that a milonga can take place anywhere in the world, and its form remains unchanged, it is, however, aimed at a limited audience with a sophisticated taste. These events are definitely conducive to social contacts, because of the trend which has developed among dancers of travelling to milongas, which undoubtedly widens the circle of friends. Attraction of tango dancing was related to travelling, often to a different cultural zone, which was emphasised by 8.3% of people. Dancers search for this type of event not only in their own cities, but also outside their own country. A special place for such trips, often in groups, is Argentina and Uruguay, as AT has becomes a driving force to visit the place where its heart undoubtedly lies—Buenos Aires. The followers of tango want to travel to the birthplace of the dance, which is their passion and soak up the atmosphere of the local milongas, exchange experiences and be exposed to and come face-to-face with the local culture and people for whom this dance is a reflection of their life [17].

In our own research, there were interesting statements from some women who declared that when they travelled away from home, they always had their dancing shoes with them and looked for these types of events in the places they visited, even during business trips. Among the respondents there were also those whose main purpose for travelling abroad was to participate in prestigious milongas. The respondents also enjoyed festivals and marathons, although the latter, in particular, require the participants to have very good physical condition and stamina.

### 4.6. Influence of Dance on the Dancers’ Mental Health

Mental health is a dynamic state of internal balance that enables individuals to use their skills in harmony with universal social values [5]. Of the women studied 14.7% noticed that AT dancing influenced their personal development. Additionally, 6.4% of the dancers said that their quality of life had increased. AT has a big influence on the human psyche and helps to restore balance in line with physiological and mental processes. It increases abstract thinking and self-control, relaxes, evokes inner peace and a feeling of calmness, and at the same time does not limit creativity. For 6.4%, tango was so attractive that it became a job, something the dancing women also spoke proudly about.

Physical activity acts as an antidepressant and helps to maintain our so-called mental well-being. It also helps to improve our emotional state, through e.g., a reduction in anxiety states [16]. As many as 42.2% of the dancers indicated that tango has a good effect on their mental state, making them, above all, happy. The women also wrote about difficult personal experiences that, through tango dancing, they were able to survive. If dance has helped one person to overcome depression, forget about the pain of splitting up with a partner, or survive difficult moments in their life, there is no doubt that it is a valuable and noteworthy way of spending time and a real help in wrestling with the difficult problems in everyday life.

In this research it was found that several women (6.4%) described the presence of tango in their lives in such a way that indicated that they were addicted to this type of physical activity. According to the WHO [34], addiction is now considered a neurobiological disease, defined as the compulsion to use a substance that is addictive together with the occurrence of withdrawal symptoms when it is stopped. The concept of addiction was then extended to ‘pathological’ behaviours such as exercise addiction. Results of the research [35] strongly suggest that dancing can be addictive. Despite the fact that dance is a form of physical activity, it is difficult not to be aware that it does differ from ordinary physical exercise. The differences include the specific nature of the tango dancing environment, the elegance of the costumes, music, sensuality stimulated by close contact with dance partners and ‘curiosity of the unknown’ when changing partners during long milongas, especially when they take place in another city or country. For these reasons, it seems that AT addiction is only partially similar to exercise addiction, and has a much deeper foundation [36]. Research [37] suggests that tango addiction may exist because several of the people they surveyed met the criteria for addiction, including: feeling tense or aroused and thirsty before dancing, pleasure and/or relief while dancing, tolerance characterised by the need to spend more time dancing and finally physical withdrawal symptoms in the absence of dancing. Why some dancers become addicted to tango and what the exact basis of this addiction is, remains unknown, but possibly several of the powerful and lasting positive effects of tango dancing may be able to explain it. These include: feelings of pleasure with a desire for repetition, self-esteem, reduced stress, and the health and fitness benefits. Compared to the relatively negligible negative effects, which may be, for example, disturbances in the circadian rhythm, the positive effects of dancing the tango seem to be much stronger and this was also shown in our research.

Dancing is an intercultural phenomenon that brings together people who share the same passion. AT creates universality representing life, ethical and aesthetic values and becomes an opportunity to distance the experiences of the individual in favour of the experiences of the partnership and become part of a community [38]. It creates a space for social, often intercultural meetings, recreation, relaxation and a feeling of pleasure. Our research has shown that the individual benefits that dance has brought into the lives of dancing women are important to their physical and mental health, and thus have a decisive influence on their quality of life.

## 5. Conclusions

The tango group compared to sedentary group is characterised by a better BMI index. The tango compared to sedentary group did not differ in the duration of menstrual bleeding, the degree of pain during menstruation, the regularity of menstruation, the number of regular monthly cycles per year, and amenorrhea. Intermenstrual spotting was more common in dancers. As it has been demonstrated through regression, the frequency of missed periods was not increased by any of the assessed aspects related to dance.

In almost sixty per cent of female dancers, milongas caused disturbances in circadian rhythms including, first and foremost, extreme fatigue, drowsiness and nonrestorative sleep. As it has been demonstrated through regression, the frequency of disturbances in circadian rhythms was not increased by any of the assessed aspects related to dance.

Answering the question about the influence of tango on personal life, a vast majority mentioned only positive aspects, most frequently: well-being, good mood, happiness, passion; health benefits, body care, “energy” and better social life, social contacts.

This study shows that AT dancing is beneficial for the physical and mental health of the women studied with only a minimal negative impact, mainly concerned with circadian rhythm disturbances in the form of fatigue, drowsiness and nonrestorative sleep. There was no impact on the women’s monthly cycle due to long dancing sessions over many years and participation in milongas and marathons, which indicates the beneficial effect of this form of physical activity on women’s health, while simultaneously safeguarding reproductive functions. Certainly, such a way of spending free time can be recommended to promote physical, mental and emotional health, as well as to satisfy the social need to ‘be with people’ and seek an escape from everyday problems.

## Figures and Tables

**Table 1:** Age and anthropometric parameters of participants.

	Median		Minimum		Maximum		U	*p*
	Sedentary	Tango	Sedentary	Tango	Sedentary	Tango		
Age [years]	36.0	37.0	23.0	22.0	54.0	51.0	4972.0	0.097
Height [cm]	167.0	165.0	148.0	153.0	185.0	183.0	5050.0	0.137
Body weight [kg]	68.0	58.0	44.0	45.0	120.0	85.0	3029.0	<0.001
BMI [kg/m^2^]	24.0	20.8	17.2	18.0	37.5	31.6	2803.0	<0.001

**Table 2:** Involvement in AT; SD—standard deviation, Min—minimum, Max—maximum.

	Mean	Median	SD	Min	Max
Number of years of AT dancing	7.3	7.0	5.1	0.5	23.0
Number of hours per week of AT dancing	7.8	6.0	6.5	0.0	30.0
Participation in marathons/festivals up to the end of the survey	18.2	12.0	19.8	0.0	100.0
Number of marathons/festivals where travelling was necessary	12.8	7.0	16.4	0.0	70.0
Number of workshops in which the dancers took part	24.9	8.0	47.3	0.0	230.0
Length of time spent at workshops [hours]	8.1	4.0	12.9	0.0	80.0

**Table 3:** Characteristics of the menstrual cycle—parametric value.

	Median		Minimum		Maximum		U	*p*
	Sedentary	Tango	Sedentary	Tango	Sedentary	Tango		
Number of days of monthly bleeding	5.0	5.0	3.0	3.0	7.0	8.0	5528.0	0.654
Degree of pain during menstrual bleeding	5.0	6.0	1.0	0.0	10.0	10.0	5364.5	0.426
Number of regular monthly cycles in the last year	12.0	12.0	7.0	1.0	14.0	12.0	5295.5	0.255

**Table 4:** Characteristics of the monthly cycle—nonparametric value; N—number of dancers, %—percentage of dancers, chi—test value, *p*—level of statistical significance.

		Sedentary		Tango		chi	*p*
		N	%	N	%		
Spotting between consecutive monthly cycles	Yes	5	4.8	14	12.8	4.32	0.038
Regularity of monthly cycles	Every 24 days	4	3.8	12	11.0	4.59	0.101
	25–31 days	87	82.9	87	79.8		
	>31 days	14	13.3	10	9.2		
Pain during menstrual bleeding	No pain	15	14.3	27	24.8	4.11	0.128
	Pain at the beginning	81	77.1	76	69.7		
	Pain throughout	9	8.6	6	5.5		
Disappearance of the monthly cycle	Never	68	64.8	77	70.6	4.11	0.128
	Less than 3 months	24	22.9	20	18.4		
	Between 3 and 6 months	7	6.7	8	7.3		
	More than 6 months	6	5.7	4	3.7		
Use of hormonal contraception	Yes	12	11.4	20	18.3	2.01	0.156

**Table 5:** Disturbances in the circadian rhythm after participating in a milonga and the percentage of women who took a break from the intensity of AT; N—number of dancers who declared a disturbance, %—percentage of dancers.

		N	%
Has participation in a milonga disturbed your circadian rhythm?	Yes	65	59.6
Type of disturbance	Extreme fatigue, drowsiness	40	36.7
	Nonrestorative sleep	32	29.4
	Inability to focus attention	22	20.2
	Difficulty in falling and staying asleep	18	16.5
	Headaches	12	11.0
	Irritability and depressed mood	11	10.1
	Appetite and gastrointestinal disorders	10	9.2
	Disorientation	8	7.3
	Bad mood	1	0.9

**Table 6:** Positive and negative changes in the personal lives of dancers caused by engaging in the physical activity of AT dancing; N—number of female dancers, %—percentage of female dancers.

	N	%
Positive Changes		
Well-being, good mood, happiness, passion	46	42.2
Health benefits, body care, “energy”	26	23.9
Better social life, social contacts	23	21.1
Personal development	16	14.7
An increase in the number of long journeys	9	8.3
Improvement in the quality of life	7	6.4
Tango has become a profession, a job	7	6.4
Positive effect on the relationship with a partner, or a change of partner	3	2.8
Negative changes		
Less sleep, disturbances in the circadian rhythm	11	10.1
Tango has reduced the time available for other life activities	7	6.4
Discomfort, tiredness, pain	2	1.8
No response	20	18.3

## Data Availability

The datasets used and/or analyzed during the current study are available from the corresponding author on reasonable request.

## Appendix A. The Author’s Own Survey

Dear Dancers of the Argentine Tango,

I would kindly ask you to take part in an anonymous survey which aims to check whether tango dancing, and this includes participation in long-lasting milongas and/or marathons which are connected with considerable physical exertion, does lead to disorders of the monthly cycle in women who practise this form of activity. Additionally, in the final question, please briefly describe how this type of physical recreation has influenced your personal life. Please give honest answers reflecting your real feelings.

1. Year of birth
2. Height [cm]
3. Body weight [kg]
4. How many years have you been dancing the tango?
5. How much time do you spend weekly dancing the tango [h]?
6. Does participation in milongas disturb the rhythm of your next 24 h? 1—YES, 2—NO
7. If you answered YES to the previous question, please indicate how your daily rhythm is disturbed (you can choose several answers):
inability to focus
extreme fatigue, drowsiness
appetite and gastrointestinal disorders
bad mood
confusion
irritability and depressed mood
headache
difficulty falling asleep and staying asleep
nonrestorative sleep
8. How many marathons / festivals have you participated in so far?
9. For how many marathons and festivals in which you participated did you have to travel to another city, country/continent?
10. Not counting those which were part of festivals, how many workshops have you participated in?
11. Enter the duration of such workshops in hours.
12. Do you take breaks from the intensity of tango dancing? 1—YES, 2—NO
13. Are you menstruating? 1—YES, 2—NO
14. If NO, what is the reason?
15. Are you currently using hormonal contraception? 1—YES, 2—NO
16. Are your monthly cycles regular, that is, every month? 1 -YES, 2—NO
How often do you menstruate?
1—every 24 days,
2—25–31 days,
3—over 31 days
17. How many days EXACTLY does your period last?
18. How many regular monthly cycles have you had in the last calendar year?
19. Are you periods painful?
1—painless
2—painful at the beginning
3—painful during the whole time
20. Please rate the degree of pain you feel during your period on a scale from 0 to 10 (0- no pain, 10—maximum pain)
21. Have you ever missed your period for a long time after a period of regular bleeding? How long did it last?
1—less than 3 months,
2—between 3 and 6 months,
3—over 6 months,
4—there has never been such a situation
22. Do you have spotting between periods? 1—YES, 2—NO
23. Please describe briefly, if and how Argentine tango dancing has influenced your life?

## Appendix B. Analyses of Spearman’s Rho Correlation between Amenorrhea and Predictors; * *p* < 0.05; ** *p* < 0.01

	**Disappearance of Monthly Cycle**	**No. of Years Dancing AT**	**No. of Hours Dancing AT Weekly**	**BMI [kg/m^2^]**	**Does Participation in Milongas Affect Your Daily Rhythm**	**No. of Marathons/Festivals**	**Journeys to Milongas away from Home Town**	**No. of Workshops Attended by the Dancers**
No. of years dancing AT	−0.03							
No. of hours dancing AT weekly	0.03	0.19 *						
BMI [kg/m^2^]	0.14 *	−0.09	0.02					
Does participation in milongas affect your daily rhythm	0.03	0.01	0.10	−0.19 *				
No. of marathons/festivals	−0.10	0.70 **	0.22 *	−0.17	0.15			
Journeys to milongas away from home town	0.07	0.28 **	0.22 *	−0.12	0.11	0.47 **		
No. of workshops attended by the dancers	0.01	0.59 **	0.44 **	−0.19 *	0.14	0.60 **	0.34 **	
Break from the intensity of dancing tango	−0.03	0.17	−0.09	−0.01	0.04	0.220 *	0.03	0.12

## Appendix C. Results for Individual Predictors of Amenorrhea. Multiple Ordinal Regression Analysis (PLUM)

	**Regression Rate (95% Confidence Intervals)**	**Wald Statistics**	***p***
Age [years]	0.01 (−0.06–0.08)	0.02	0.879
BMI [kg/m^2^]	0.12 (−0.06–0.30)	1.66	0.197
No. of years dancing AT	−0.04 (−0.14–0.06)	0.59	0.444
No. of hours dancing AT weekly	0.01 (−0.06–0.08)	0.07	0.794
Does participation in milongas affect your daily rhythm	0.15 (−0.72–1.02)	0.11	0.742
Journeys to milongas away from home town	0.78 (−0.51–2.06)	1.41	0.235
Break from the intensity of dancing tango	−0.10 (−0.98–0.77)	0.05	0.816

## Appendix D. Results for Predictors of Disturbances in Circadian Rhythm after Milongas. Multiple Logistic Regression Analysis

	**Odds Ratio** **(95% Confidence Intervals)**	**Wald Statistics**	***p***
Age [years]	1.03 (0.97–1.11)	0.93	0.335
BMI [kg/m^2^]	0.85 (0.71–1.02)	2.93	0.087
No. of years dancing AT	0.95 (0.87–1.04)	1.11	0.291
No. of hours dancing AT weekly	1.05 (0.98–1.12)	1.72	0.190
Journeys to milongas away from home town	1.40 (0.47–4.14)	0.36	0.549
Break from the intensity of dancing tango	1.21 (0.52–2.80)	0.19	0.659
